# Research and Remedies

**Published:** 1914-03-28

**Authors:** 


### RESEARCH AND REMEDIES.

### Some Edinburgh Cancer Statistics.

Cancerous growths of the upper end of the œsophagus are not very common, and reliable statistics based on a large number of cases are therefore not easy to obtain. Dr. A. Logan Turner has analysed in the *Journal of Laryngology, Rhinology, and Otology* a series of sixty-eight such cases at Edinburgh, and draws the following conclusions: Carcinoma of the upper end of the œsophagus is more common in women than in men. It occurs in women at an earlier age than in men, and it invades the hypopharynx more frequently in women. The average duration of the disease is longer in women than in men, and in older than in younger patients. The average duration of the disease is shorter when the hypopharynx is involved than when it is limited to the upper end of the œsophagus. Laryngoscopy is an essential part of the examination in all cases suspected of carcinoma of the œsophagus; but bougies should not be employed for diagnosis. A combination of $x$-rays and œsophagoscopy furnishes the most complete information regarding the nature of the disease, its situation, and the length of the gullet involved. Finally, when a piece of the suspected tissue is removed for microscopic examination, and no evidence of carcinoma is found, the existence of a simple stricture must not be inferred.

### Lingual Gymnastics.

At a meeting of the Laryngological Section of the Royal Society of Medicine a woman was shown who possesses the power to pass her tongue behind her soft palate, and can thus identify for herself the ends of the Eustachian tubes and the inferior turbinals. One of the surgeons present recalled a case of atrophic rhinitis, in which the patient had learnt to get rid of the crusts by means of the tongue; and two or three others alluded to the possibility of committing suicide by this means. It appears that slaves on the slave-ships were known to practise this lingual method of self-suffocation in order to obtain release from their wretchedness.

